# Hypothalamic-Pituitary-Adrenal Axis Abnormalities in Response to Deletion of 11β-HSD1 is Strain-Dependent

**DOI:** 10.1111/j.1365-2826.2009.01899.x

**Published:** 2009-11

**Authors:** R N Carter, J M Paterson, U Tworowska, D J Stenvers, J J Mullins, J R Seckl, M C Holmes

**Affiliations:** * Endocrinology Unit, Centre for Cardiovascular Science, Queen’s Medical Research Institute, University of EdinburghEdinburgh, UK; † Molecular Physiology, Centre for Cardiovascular Science, Queen’s Medical Research Institute, University of EdinburghEdinburgh, UK

**Keywords:** 11β-HSD1, knockout mice, glucocorticoids, mouse strain, HPA axis

## Abstract

Inter-individual differences in hypothalamic-pituitary-adrenal (HPA) axis activity underlie differential vulnerability to neuropsychiatric and metabolic disorders, although the basis of this variation is poorly understood. 11β-Hydroxysteroid dehydrogenase type 1 (11β-HSD1) has previously been shown to influence HPA axis activity. 129/MF1 mice null for 11β-HSD1 (129/MF1 HSD1^−/−^) have greatly increased adrenal gland size and altered HPA activity, consistent with reduced glucocorticoid negative feedback. On this background, concentrations of plasma corticosterone and adrenocorticotrophic hormone (ACTH) were elevated in unstressed mice, and showed a delayed return to baseline after stress in HSD1-null mice with reduced sensitivity to exogenous glucocorticoid feedback compared to same-background genetic controls. In the present study, we report that the genetic background can dramatically alter this pattern. By contrast to HSD1^−/−^ mice on a 129/MF1 background, HSD1^−/−^ mice congenic on a C57Bl/6J background have normal basal plasma corticosterone and ACTH concentrations and exhibit normal return to baseline of plasma corticosterone and ACTH concentrations after stress. Furthermore, in contrast to 129/MF1 HSD1^−/−^ mice, C57Bl/6J HSD1^−/−^ mice have increased glucocorticoid receptor expression in areas of the brain involved in glucocorticoid negative feedback (hippocampus and paraventricular nucleus), suggesting this may be a compensatory response to normalise feedback control of the HPA axis. In support of this hypothesis, C57Bl/6J HSD1^−/−^ mice show increased sensitivity to dexamethasone-mediated suppression of peak corticosterone. Thus, although 11β-HSD1 appears to contribute to regulation of the HPA axis, the genetic background is crucial in governing the response to (and hence the consequences of) its loss. Similar variations in plasticity may underpin inter-individual differences in vulnerability to disorders associated with HPA axis dysregulation. They also indicate that 11β-HSD1 inhibition does not inevitably activate the HPA axis.

Alteration in the regulation of the hypothalamic-pituitary-adrenal (HPA) axis has been implicated in a number of disease states, ranging from metabolic disorders (1) to psychiatric conditions such as depression (2, 3) and post-traumatic stress disorder (4, 5). Although the relationship between the different HPA states to the pathophysiology of these disorders is unclear, reports of the efficacy of glucocorticoid lowering therapies in metabolic syndrome and in depression (6–9) suggest a role in pathogenesis and/or its maintenance. Clearly, the genetic mechansims that underpin individual differences in HPA axis function are of considerable importance.

HPA axis output is normally measured in terms of plasma concentration of glucocorticoids such as corticosterone (rodent) and cortisol (human). However, of equal or greater importance is tissue sensitivity to the steroids. Genetic variations in the two nuclear receptors for glucocorticoids, mineralocorticoid receptor (MR) and glucocorticoid receptor (GR), associate with the risk of cardio-metabolic and neuropsychiatric disease (10–13). In addition, the effective concentration of glucocorticoids within cells can be amplified by conversion of inactive steroids, 11-dehydrocorticosterone and cortisone, into active glucocorticoids in tissues expressing 11β-hydroxysteroid dehydrogenase type 1 (11β-HSD1). *In vivo*, 11β-HSD1 is the sole enzyme capable of regeneration of active 11-hydroxy steroids from their inactive keto forms (14). 11β-HSD1 is also expressed in many peripheral sites, including the liver, adipose, and bone (15, 16). 11ß-HSD1 is also highly expressed in the brain, including the cortex, hippocampus and cerebellum (17, 18), as well as the paraventricular nucleus of the hypothalamus (PVN) (19), where changes in enzyme activity associate with alterations in HPA axis function (20). The ability of 11β-HSD1 to amplify glucocorticoids locally, and its expression in areas known to be relevant to glucocorticoid regulation of HPA axis control, suggest that the enzyme may take part in the overall regulation of HPA output, in addition to modulating local steroid concentrations.

Characterisation of 11β-HSD1 knockout (HSD1^−/−^) mice on a mixed genetic background (129/MF1) showed HPA characteristics consistent with a role for 11β-HSD1 in HPA regulation. As anticipated by the increased clearance of glucocorticoids in these mice (i.e. the result of the loss of regeneration of steroids by 11β-HSD1 in liver and other peripheral organs), 129/MF1 HSD1^−/−^ mice have enlarged adrenals to compensate (14, 21). However, the null mice also showed elevated morning (nadir) basal plasma corticosterone and adrenocorticotrophic hormone (ACTH). Corticosterone rose to peak levels earlier in the day in 129/MF1 HSD1^−/−^ mice (14, 21). 129/MF1 HSD1^−/−^ mice also showed an increased early rise in plasma corticosterone in response to brief restraint (21), and prolonged elevation of both ACTH and corticosterone after termination of restraint (21). Based on these data, and observations of reduced 11β-HSD1 in the hippocampus in obese rats with attenuated HPA feedback control (22), it was hypothesised that 11β-HSD1 contributes to glucocorticoid negative-feedback control of the HPA axis (23). In an effort to further study these initial observations, MF1/129 HSD1^−/−^ mice were repeatedly back crossed onto a C57Bl/6J background to reach genetic homogeneity of background. On this background, the metabolic and hippocampus-associated cognitive effects seen on the mixed 129/MF1 background are recapitulated (24, 25).

In the present study, we examined the HPA axis in C57Bl/6J HSD1^−/−^ mice and show that basal plasma corticosterone levels in HSD1^−/−^ mice on the C57Bl/6J background, as opposed to that observed in 129/MF1 mice, are similar to controls. To investigate a mechanism by which this apparent strain difference may be explained, we determined GR, MR and corticotrophin-releasing factor (CRF) mRNAs in the brain and performed a dexamethasone suppression test to determine GR-mediated feedback sensitivity.

## Materials and methods

### Animals

Adult C57Bl/6J HSD1^−/−^ male mice were generated by back-crossing a minimum of 10 generations onto a C57Bl/6J (Harlan, Essex, UK) background from the original MF1/129 HSD1^−/−^ line (14). Controls were male C57Bl/6J mice from the same source, maintained and bred within the animal house designated C57Bl/6J HSD1^+/+^. Animals were housed singly for at least 1 week prior to experiments and were age-matched (between 3–6 months of age). The light/dark cycle was 12 : 12 h (lights on 07.00 h). Animals were given standard chow and water *ad lib*, and all studies were carried out to the highest standards under the aegis of the United Kingdom Animals Scientific Procedures Act, 1986. Previously reported data from HSD1^−/−^ male mice on other backgrounds comprised: 129 mice (26) and 129/MF1 mice (14, 21).

### Circadian measurements

To determine activity of the HPA axis at various times during the circadian cycle, C57Bl/6J HSD1^−/−^ and HSD1^+/+^ mice were sacrificed without prior disturbance at 07.00 h, 13.00 h and 19.00 h. Sacrifice was performed by direct decapitation, and trunk blood was collected into EDTA-coated microvette tubes (Sarstedt, Numbrecht, Germany). Tubes were put on ice until all samples were collected. Tubes were then spun 10 min at 2300 ***g*** in a chilled microcentrifuge. Supernatants (plasma) were stored at −20 °C until analysed.

### Restraint stress

All procedures were carried out between 08.00–10.00 h. C57Bl/6J HSD1^−/−^ and HSD1^+/+^ mice were removed from their home cage to a nearby procedure room. Restraint was carried out for 10 min by placing the mouse into a 50 ml Falcon tube (Greiner Bio-One, Gloucestershire, UK), which was modified to allow the tail to protrude out the back with a breathing hole at the front. Mice were either sacrificed immediately or returned to their home cage. Separate groups of mice were sacrificed 10, 45 or 90 min after the start of restraint. An additional group of mice was sacrificed without exposure to restraint within 1 min after removal from their home cage to obtain basal corticosterone levels. Killing and blood collection were performed as above.

### Analysis of plasma hormones

Analysis of plasma corticosterone and ACTH was performed as described previously (21). In short, corticosterone was measured by radioimmunoassay using ^3^H -corticosterone label, and a polyclonal anti-corticosterone antibody (kind gift of Dr C. J. Kenyon, Edinburgh). ACTH was measured by an enzyme-linked immunosorbent assay kit using a monoclonal anti-human ACTH antibody designed against regions of ACTH that are 100% conserved in the mouse (Biomerca, Newport Beach, CA, USA).

### Adrenal measurements

Left adrenals were removed at sacrifice from C57Bl/6J HSD1^−/−^ and HSD1^+/+^ mice, and placed immediately into 4% paraformaldehyde (Sigma-Aldritch, Poole, UK). Twenty-four hours post fixation, adrenals were cleaned of any attached fat by manual dissection. Adrenals were then weighed on a microbalance. Body weight of mice was taken prior to sacrifice.

### *In situ* hybridisation

*In situ* hybridisation was performed as described previously (21). All *in situ* experiments were performed on fresh frozen brains collected after decapitation from unstressed animals at the nadir of the corticosterone rhythm (08.00 h). Cryostat cut sections (10 μm) were collected at the level of the hypothalamic paraventricular nucleus and the dorsal hippocampus. CRF, GR and MR mRNAs were all detected by riboprobe based *in situ* hybridisation autoradiography. Briefly, plasmids containing fragments of cDNA for rat GR (673 bp; *Pst*1-*Eco*RI fragment), MR (513 bp; *Eco*RI fragment) and CRF (518 bp; *Pvu*II-*Bam*HI fragment) were used as templates to transcribe ^35^S-UTP radiolabelled antisense riboprobes. After hybridisation and stringent washes, the sections were exposed to autoradiographic film (XAR-5; Kodak, Kemel Hempstead, UK). Specific optical density measurements of different regions of the brain were obtained after subtraction of background density (obtained over white matter), average from eight to ten measurements/area per section, three sections per anatomical area/mouse using computer-driven densitometry (MCID; Interfocus, Cambridge, UK). CRF was measured exclusively in the PVN, MR in the dorsal hippocampus (CA1, CA3 and dentate gyrus), and GR was measured in the PVN and the three divisions of the dorsal hippocampus.

### Preparation of RNA from pituitaries

Pituitaries were obtained from freshly killed mice, and immediately frozen on dry ice. RNA was obtained from each single pituitary by homogenisation in 300 μl Trizol reagent (Gibco BRL, Paisley, UK) on ice (4 °C) using a hand held glass homogeniser. Homogenates were transferred to a 1.5-ml eppendorf and incubated at room temperature for 5 min. To each, 30 μl of chloroform was added, contents mixed by inversion, and then spun at 4 °C at 9300 ***g*** for 10 min in a table top microcentrifuge. The top layer containing RNA was removed, and transferred to a new eppendorf tube. One volume of 70% ethanol was added, contents mixed, and RNA purified using the RNeasy purification kit (Qiagen, GmbH, Hilden, Germany). Samples were transferred to an RNEasy column and spun for 15 s at 15 700 ***g*** at room temperature. Seven hundred microlitres of buffer RW1 was added to the column, and the column was spun for another 15 s at 15 700 ***g***. Five hundred microlitres of buffer RPE was added and spun for 15 s at 15 700 ***g*** Another 500 μl of buffer RPE was added and spun for 2 min at 15 700 ***g***. The column was spun once more (dry) for 1 min at 15 700 ***g*** to remove trace wash solution. Column bound RNA was eluted from the column by adding 30 μl RNAse free water followed by a spin for 1 min at 15 700 ***g***. RNA was stored at −80 °C until ready for cDNA synthesis.

### cDNA synthesis from pituitary RNA

cDNA from pituitary RNA was synthesised using Invitrogen cDNA synthesis reagents (Invitrogen, Carlsbad, CA, USA). Approximately 0.5 μg of RNA was heated to 70 °C for 10 min then placed on ice. RNA was incubated in a 20-μl reaction containing 1st-Strand reaction buffer, 500 μm dNTPs, 2.5 mm MgCl_2_, 5 mm dithiothreitol, RNAse inhibitor, 300 ng random primers, and Superscript III reverse transcriptase. The mixture was incubated at 25 °C for 5 min, followed by 60 min at 50 °C. The reaction was terminated by incubation at 80 °C for 15 min. cDNA was stored at −20 °C until ready for use in a real-time polymerase chain reaction (PCR) assay. A reaction, which excluded the Superscript III reverse transcriptase, was performed for each sample, as a negative control for the real-time PCR reaction.

### Quantitative real-time PCR for quantification of GR mRNA

Real-time PCR was performed using the Light Cycler 480 PCR machine (Roche Diagnostics, Mannheim, Germany). Polymerase, buffer and dNTPs were provided using reagents from the Light Cycler 480 Probes Master kit (Roche Diagnostics). GR specific primers/probe were designed from mouse cDNA sequence data and synthesised by Eurogentec S. A. (Seraing, Belgium). Forward primer: 5′-CCC TGG AAT GAG ACC AGA TG-3′, Reverse primer: 5′-GGT AAT TGT GCT GTT CTT CCA C-3′, Probe: 5′-CTG CCT GGT GTG CTC CGA TGA AGC-3′. The probe for GR is labelled with the 5′fluorescence reporter Yakima Yellow, the 3′ quencher BHQ-1, and detected on the VIC channel of the Cycler. Quantification of GR was expressed relative to an internal control, 18S. A standard 18S specific primers/probe mix was used (18S Taqman gene expresion assay, Hs99999901_s1; Applied Biosystems, Foster City, CA, USA). The 18S probe was detected on the FAM channel. Primers were used at a 6 pmol/reaction, and probes at 2 pmol/reaction. Relative values of both GR and 18S were calculated by comparison with a standard curve. Samples to be used to generate standard curves were prepared: 2 μl of pituitary cDNA from each individual animal was mixed together. This mixture was then serially diluted with PCR grade water to produce standards with relative concentrations of 1 (The original mixture), 1 : 2, 1 : 4, 1 : 8, 1 : 16, 1 : 32, 1 : 64, 1 : 128 and 1 : 256. Individual pituitary cDNA samples and their corresponding negative control samples (Superscript III reverse transcriptase excluded), were diluted 1 : 20 in PCR grade water. Two microlitres of 1 : 20 diluted cDNA was mixed with 5 μl master mix, 0.5 μl primer/probe mix and 2.5 μl H_2_O. Reactions were performed in triplicate for GR and 18S on the Cycler. PCR was performed using cycling parameters of 50 °C for 2 min, 95 °C for 10 min, 40 cycles of 95 °C for 15 s, and 60 °C for 1 min. Relative cDNA quantities for both GR and 18S were derived from each reaction by comparison with the standard curve using the absolute quantification/second derivative max calculation method on the Light Cycler 480 System. Amplification of negative control samples occurred after many more cycles (> 10) than for experimental samples and were excluded from the analysis. GR/18S ratios were obtained for each individual, and these ratios were used for presentation and statistical analysis of the data.

### Dexamethasone suppression test

To assess dexamethasone feedback regulation of HPA activity, we performed a modified version of the dexamethasone suppression test. Mice of both genotypes were injected i.p. with 200 μl of vehicle [2% ethanol (BDH, Dorset, UK)/0.9% saline (Sigma-Aldritch)] at 13.00 h. Venesection of the tail was used to obtain blood samples from these animals at 19.00 h the same day (near peak of circadian rise in plasma corticosterone). Blood was collected and plasma prepared as above, and processed for corticosterone measurements. At 2-weekly intervals, the same mice were injected with 2 μg/kg or 10 μg/kg dexamethasone (Sigma-Aldritch) in approximately 200 μl vehicle at 13.00 h, followed by tail blood sampling at 19.00 h.

### Statistical analysis

Adrenal weight, and gene expression data obtained from unstressed animals were analysed by an independent samples t-test to determine an effect of genotype. Plasma corticosterone and ACTH data from stress experiments were analysed by two-way analysis of variance (anova) for the effects of time and genotype. Corticosterone data from dexamethasone suppression tests were analysed by a repeated measures design two-way anova. Post-hoc analysis was performed using the Tukey’s honestly significant difference test. P < 0.05 was considered statistically significant.

## Results

### Adrenal weight is increased in C57Bl/6J HSD1^−/−^ mice

**Fig. 1:**
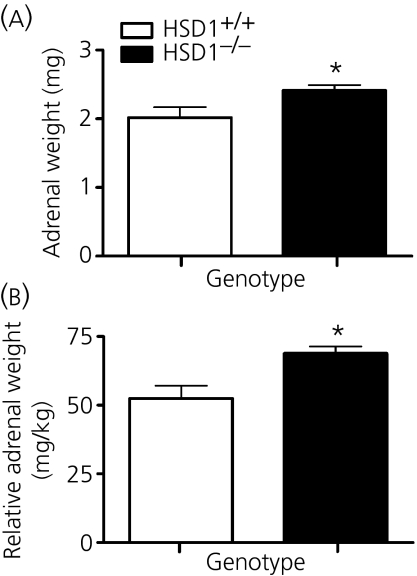
Adrenal glands are larger in adult male C57Bl/6J 11βHSD1^−/−^ mice compared to C57Bl/6J controls. (a) Absolute left side adrenal weights (mg) are larger in C57Bl/6J 11βHSD1^−/−^ mice (*P < 0.05, compared to controls). (b) Left adrenal weight relative to body weight expressed in mg/kg are larger in C57Bl/6J 11βHSD1^−/−^ mice (*P < 0.05, compared to controls). Values are the mean ± SEM.

Adrenal mass was significantly higher in C57Bl/6J HSD1^−/−^ mice compared to control C57Bl/6J HSD1^+/+^ mice, by approximately 20% (Fig.1a) (t = 2.19, P = 0.042; n = 10 per group). The adrenal enlargement was also observed when adrenal weights were expressed relative to body weight (Fig. 1b) (t = 3.12, P = 0.006; n = 10), reiterating the findings on the 129/MF1 background (26). Body weights were not significantly different on the C57Bl/6J background (HSD1^+/+^ 38.8 +/− 1.5 g, HSD1^−/−^ 35.4 +/− 0.73 g; n = 10).

### Circadian rhythms of plasma corticosterone and ACTH are unaltered in C57Bl/6J HSD1^−^/ ^−^ mice

We have previously shown that MF1/129 HSD1^−/−^ mice have elevated nadir (08.00 h) and an earlier diurnal rise in plasma corticosterone levels compared to MF1/129 HSD1^+/+^ mice. We therefore investigated the circadian profile of plasma corticosterone in both C57Bl/6J HSD1^−/−^ and C57Bl/6J HSD1^+/+^ mice.

**Fig. 2:**
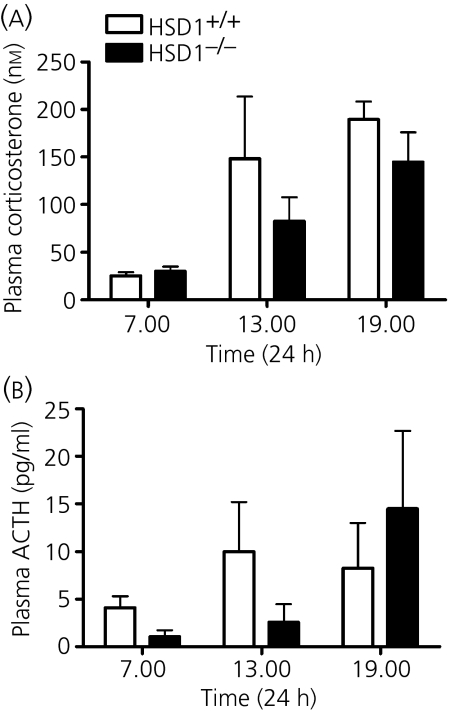
Circadian profile of hypothalamic-pituitary-adrenal axis activity is normal in adult male C57Bl/6J 11βHSD1^−/−^ mice compared to C57Bl/6J control mice. (a) Plasma levels of corticosterone (nm) secreted at 07.00, 13.00 and 19.00 h. Levels at 19.00 h were elevated from basal levels at 06.00 h in both genotypes. There were no differences between genotypes at any time point. (b) Plasma levels of adrenocorticotrophic hormone (ACTH) (pg/ml) secreted at 07.00, 13.00 and 19.00 h. There were no differences between genotypes at any time point, nor was there significant variation throughout the day. Values are the mean ± SEM.

Although there was a clear difference between basal levels (07.00 h) and peak levels (19.00 h) in all mice, two-way anova for the effects of time and genotype indicated an effect of time (F_2,24_ = 9.55, P = 0.001), but not genotype (F_1,24_ = 1.77, P = 0.196), nor any interaction between genotype and time (F_2,24_ = 0.637, P = 0.537) (Fig. 2a). Therefore, there is no evidence of either increased basal plasma corticosterone or earlier rise to peak levels in C57Bl/6J HSD1^−/−^ mice. Similarly, plasma levels of ACTH did not differ between C57Bl/6J HSD1^−/−^ and C57Bl/6J HSD1^+/+^ mice (two-way anova). There was no indication of elevated nadir ACTH in C57Bl/6J HSD1^−/−^ mice compared to C57Bl/6J HSD1^+/+^ mice (Fig. 2b), contrasting with findings previously observed in MF1/129 HSD1^−/−^ mice (21).

### Altered plasma corticosterone, but not ACTH, response to restraint stress in C57Bl/6J HSD1^−/−^ mice

We have previously shown that MF1/129 HSD1^−/−^ mice have an exaggerated stress-induced rise in plasma corticosterone levels, and a retarded return to baseline of both corticosterone and ACTH compared to strain controls. In the present study, we determined stress induced levels of plasma corticosterone in C57Bl/6J HSD1^−/−^ and C57Bl/6J HSD1^+/+^ mice.

**Fig. 3:**
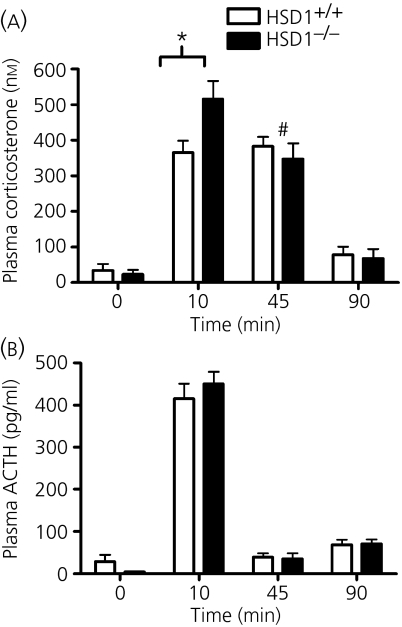
Initial rise in hypothalamic-pituitary-adrenal axis activity in response to 10-min restraint is increased in C57Bl/6J 11βHSD1^−/−^ mice, but return to baseline after termination of restraint is normal, compared to C57Bl/6J control mice. (a) C57Bl/6J 11βHSD1^−/−^ mice had higher plasma corticosterone levels than C57Bl/6J control mice after 10 min of restraint (*P < 0.05 compared to C57Bl/6J controls). Corticosterone levels after 45 min were reduced in C57Bl/6J 11βHSD1^−/−^ mice (but not C57Bl/6J controls) from levels observed after 10 min (#P < 0.05 compared to the 10-min time point). Corticosterone had reached levels indistinguishable from baseline after 90 min in mice from both genotypes. (b) Plasma adrenocorticotrophic hormone (ACTH) levels were elevated after 10 min of restraint and had returned to levels similar to baseline after 45 or 90 min. There were no differences between genotypes at any time point. Values are the mean ± SEM.

Again, basal (nadir) plasma corticosterone levels did not differ between C57Bl/6J HSD1^+/+^ and C57Bl/6J HSD1^−/−^ mice (Fig. 3a). Ten minutes of restraint led to marked elevations in plasma corticosterone in mice of both genotypes, but a significantly greater response in C57Bl/6J HSD1^−/−^ mice (P < 0.05) (Fig. 3a). These levels remained elevated over basal after 45 min, but there was a significant reduction of peak values only in the C57Bl/6J HSD1^−/−^ mice at this time-point (P < 0.05) (Fig. 3a). By 90 min, plasma corticosterone levels in both genotypes were similar to unstressed values (Fig. 3a).

Plasma ACTH levels did not differ between C57Bl/6J HSD1^+/+^ and C57Bl/6J HSD1^−/−^ mice, prior to or during the response to restraint (Fig. 3b). Ten minutes of restraint led to increased levels of plasma ACTH in mice of both genotypes, returning to baseline by 45 min (Fig. 3b). A two-way anova for the effect of time and genotype on plasma ACTH revealed no main effect of genotype (F_6,40_ = 0.034, P = 0.85), but a significant effect of time (F_6,40_ = 207.9, P < 0.001).

The normal return to baseline of plasma corticosterone and ACTH levels in response to restraint stress in C57Bl/6J HSD1^−/−^ mice is indicative of a tightly regulated HPA axis, suggesting that these mice may induce compensatory mechanisms not seen in MF1/129 HSD1^−/−^ mice. We therefore looked at expression of genes important in HPA axis regulation.

### C57Bl/6J HSD1^−/−^ null mice have altered gene expression in the paraventricular nucleus of the hypothalamus and the hippocampus

**Fig. 4:**
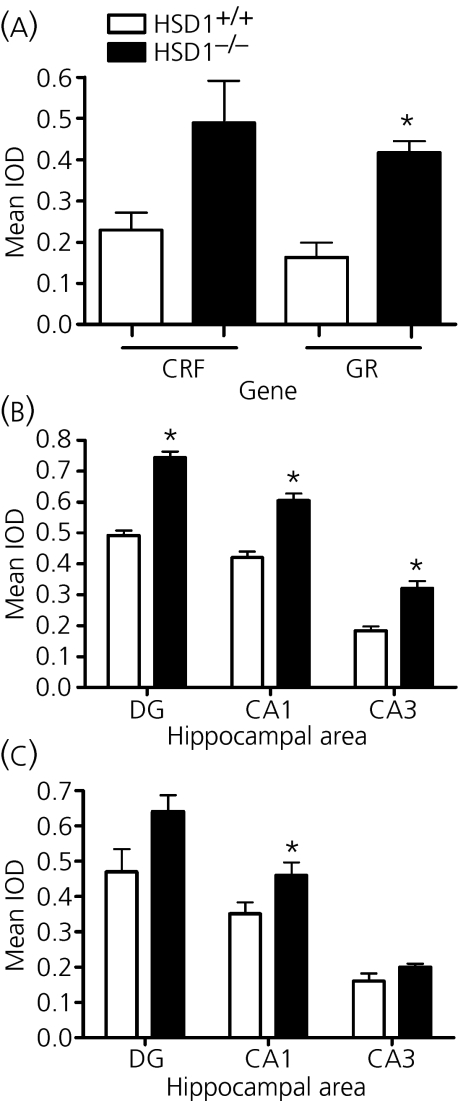
Basal (nadir) steady-state mRNA levels of corticotrophin-releasing factor (CRF), glucocorticoid receptor (GR) and mineralocorticoid receptor (MR) in HSD1^−/−^ and C57Bl/6J mice in the paraventricular nucleus (PVN) and hippocampus. *In situ* hybridisation for each study was analysed by integrated optical density (IOD) measurements of autoradiographs. (a) IOD measurements for CRF and GR mRNA in the PVN. CRF expression tends towards being increased in the PVN of HSD1^−/−^ mice (α, P = 0.057, compared to C57Bl/6J controls). GR expression is increased in the PVN of HSD1^−/−^ mice (*P < 0.05, compared to C57Bl6 controls). (b) IOD measurements for GR mRNA in the dentate gyrus (DG), CA1 and CA3 areas of the hippocampus. GR expression is increased in all areas of the hippocampus of HSD1^−/−^ mice (*P < 0.05, compared to C57Bl6 controls). (c) IOD measurements for MR mRNA in the DG, CA1, and CA3 areas of the hippocampus. MR expression is increased only in the CA1 of the hippocampus of HSD1^−/−^ mice (*P < 0.05, compared to C57Bl/6J controls). Values are the mean ± SEM.

C57Bl/6J HSD1^−/−^ mice show considerable differences in the expression of HPA relevant genes in the PVN and hippocampus relative to C57Bl/6J HSD1^+/+^ mice. In the PVN, GR mRNA expression was significantly elevated (P < 0.001) and CRF mRNA showed a tendency to be elevated (P = 0.057) in C57Bl/6J HSD1^−/−^ mice (Fig. 4a). Expression of GR mRNA in the hippocampus was also elevated in C57Bl/6J HSD1^−/−^ mice throughout all measured subfields (P < 0.001) (Fig. 4b). MR mRNA expression was significantly elevated only in the CA1 region (P < 0.05), but not in the CA3 and dentate gyrus in C57Bl/6J HSD1^−/−^ mice (Fig. 4c). By contrast, the expression of GR mRNA in the pituitary was not altered in C57Bl/6J HSD1^−/−^ mice relative to C57Bl/6J HSD1^+/+^ mice (Table 1). The up-regulation of corticosteroid receptors in areas of the brain (but not pituitary) involved in negative-feedback regulation of the HPA axis suggests that this may be an important mechanism whereby C57Bl/6J HSD1^−/−^ mice may be able to reset their axis and maintain normal basal plasma corticosterone levels. Therefore, we determined whether the C57Bl/6J HSD1^−/−^ mice have increased sensitivity to feedback, using the dexamethasone suppression test.

**Table 1:** Glucocorticoid Receptor (GR) mRNA Expression in the Pituitary of HSD1^−/−^ and HSD1^+/+^ Mice Estimated by Quantitative Real-Time Polymerase Chain Reaction (Relative to 18S Internal Control).

Genotype	GR/18S ratio (mean ± SEM)
HSD1^+/+^	0.998 ± 0.071
HSD1^−/−^	0.904 ± 0.086

### C57Bl/6J HSD1^−/−^ mice show greater sensitivity to dexamethasone suppression of peak plasma corticosterone

**Fig. 5:**
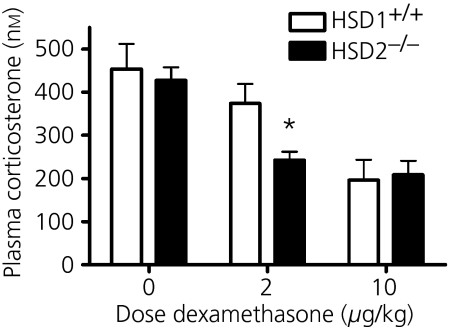
C57Bl/6J 11βHSD1^−/−^ mice are hypersensitive to dexamethasone suppression of evening plasma corticosterone compared with C57Bl/6J control mice. C57Bl/6J 11βHSD1^−/−^ mice, but not C57Bl/6J controls had reduced evening (19.00 h) plasma corticosterone after 2 μg/kg dexamethasone given i.p. 5 h previously (*P < 0.05, compared to vehicle). Plasma corticosterone was reduced in mice from both genotypes after 10 μg/kg dexamethasone. Values are the mean ± SEM.

Evening plasma corticosterone levels were suppressed by prior dexamethasone administration, but the minimal effective dose differed between C57Bl/6J HSD1^−/−^ mice and C57Bl/6J HSD1^+/+^ mice (Fig. 5). Repeated measures anova for between-subjects effect of genotype and within-subjects effect of dose revealed a main effect of dose (F = 20.2, P < 0.0001) but not genotype. Post-hoc analysis revealed that afternoon plasma corticosterone was reduced from levels observed in vehicle-injected mice with a dose of 2 μg/kg dexamethasone in C57Bl/6J HSD1^−/−^ mice, but not C57Bl/6J HSD1^+/+^ mice (Fig. 5). Plasma corticosterone was reduced from levels observed in vehicle-injected mice after a dose of 10 μg/kg dexamethasone in mice of both genotypes (Fig. 5). Hence, C57Bl/6J HSD1^−/−^ mice appear to be more sensitive to negative-feedback signals than controls.

### Summary of HPA profile of HSD1^−/−^ mice on different strain backgrounds

In addition to the data presented for HSD1^−/−^ mice congenic to C57Bl/6J, Table 2 presents a summary of various aspects of the HPA axis that we found in HSD1^−/−^ mice on a number of different strain backgrounds. These include our published data on the original 129/MF1 cross (21) and on HSD1^−/−^ congenic on the 129 background (26).

**Table 2:** Summary of Hypothalamic-Pituitary-Adrenal Axis Phenotype of HSD1^−/−^ Mice, From Different Strain Backgrounds.

Measure	129	129/MF1	C57Bl/6J
Adrenal size	↑	↑	↑
Basal AM Cort	↑	↑	↔
Basal PM Cort	ND	↔	↔
Peak Stress Cort	ND	↑	↑
Recovery Cort	ND	↑	↔
Basal AM ACTH	ND	↑	↔
Basal PM ACTH	ND	↔	↔
Peak Stress ACTH	ND	↔	↔
Recovery ACTH	ND	↑	↔
CRF mRNA (paraventricular nucleus)	ND	↓*	↑*
GR mRNA (paraventricular nucleus)	ND	↓	↑
GR mRNA (hippocampus)	↓	↔	↑
MR mRNA (hippocampus)	ND	↔	↑*

Arrows designate direction of change in HSD1^−/−^ mice relative to HSD1^+/+^ mice from the same background: ↑, higher; ↓, lower; ↔, not different. All changes are significant to P < 0.05, except those designated by an asterisk (*), which tend towards change with P < 0.1. Measurements not carried out on a particular background are indicated by ND (not done). ACTH, adrenocorticotrophic hormone; CRF, corticotrophin-releasing factor; AM Cort, morning corticosterone; PM Cort, afternoon corticosterone; GR, glucocorticoid receptor; MR, mineralocorticoid receptor.

Adrenal size is increased in HSD1^−/−^ mice on all strain backgrounds. Consistent with this, early peak stress corticosterone levels are also increased in HSD1^−/−^ mice on all backgrounds. However, although basal (early morning) corticosterone levels are increased in HSD1^−/−^ mice on the 129 and 129/MF1, they are the same as controls on the C57Bl/6J background, despite an increased adrenal size. A disturbed overall rhythm is observed in 129/MF1 HSD1^−/−^ but not C57Bl/6J HSD1^−/−^ mice. These data suggest that genetic elements, probably from the 129 strain, contribute to the altered basal plasma corticosterone of HSD1^−/−^ mice. Shut off of HPA activity after stress is also abnormal in 129/MF1 HSD1^−/−^ mice, but not in C57Bl/6J HSD1^−/−^ mice. In line with these observations are the findings regarding GR and MR expression in the brain of HSD1^−/−^ mice. 129/MF1 mice, with features consistent with impaired negative-feedback regulation of plasma glucocorticoid levels, have reduced GR expression in the PVN. Conversely, C57Bl/6J HSD1^−/−^ mice, with apparent normal plasma glucocorticoid regulation (despite loss of 11β-HSD1), have elevated GR expression in both the PVN and the hippocampus. Increased suppression by dexamethasone of HPA activity in HSD1^−/−^ mice congenic to C57Bl/6J suggests that feedback sensitivity is increased, as would be predicted from increased GR expression in feedback-sensitive sites such as the PVN and hippocampus.

## Discussion

### HPA phenotype of HSD1^−/−^ mice is dependent upon strain background

In the present study, we report that, on a strain background congenic to C57Bl/6J, no differences were observed between HSD1^−/−^ mice and HSD1^+/+^ mice in either plasma corticosterone or ACTH at the nadir of the HPA rhythm. This is in contrast to the marked hypercorticosteronemia and elevated morning ACTH previously reported in HSD1^−/−^ mice on the MF1/129 background (21). Although adrenal weight was found to be increased in size in C57Bl/6J HSD1^−/−^ mice in the present study, in line with the previous report, the extent of size difference is much less dramatic. Adrenal weights were increased approximately 70% in HSD1^−/−^ mice on the MF1/129 background (14), but only 20% in C57Bl/6J HSD1^−/−^ mice in the present study. The ability to ‘turn off’ the hormone response to a stressor is, in part, indicative of the strength of the negative-feedback signal within the brain and the anterior pituitary (27). The delayed return of both plasma corticosterone and ACTH to pre-stress levels after restraint, as seen on MF1/129 HSD1^−/−^ mice, is no longer observed in C57Bl/6J HSD1^−/−^ mice. These strain differences in HPA axis parameters contrast with the similar metabolic and cognitive phenotypes seen with HSD1^−/−^ on 129/MF1 crossed and congenic 129 and C57Bl/6J backgrounds (14, 23, 26, 28, 29).

The phenotype of elevated basal plasma corticosterone is most likely contributed to largely by genetic modifiers within the 129 strain genome. Consistent with this hypothesis is the observation that the HPA phenotype in HSD1^−/−^ mice on a pure 129 background is remarkably similar to the phenotype on the MF1/129 background, yet 11β-HSD1^−/−^ mice on a pure MF1 background show little or no HPA phenotype at all (R. Carter, M. C. Holmes, unpublished observations), including a lack of both increased adrenal size or peak corticosterone in response to stress. The 129 strain has often been compared to other strains, including C57Bl/6J, and is known to show more anxiety-related behaviours in the elevated plus-maze (30), open field (31) and light/dark test (32). One hundred twenty-nine mice also showed greater sensitivity to benzodiazepine anxiolytics (33), perhaps indicating an altered GABA system. There is some suggestion of elevated basal corticosterone in 129 relative to C57Bl/6J mice (34), although we found no evidence for this in our own comparisons (25, 26, 35). However, the importance of genetic background in determining the phenotype of transgenic animals has been recognised for some time (36–38). For example, knockout mice for the ACTH processing genes, prohormone convertase and 7B2 have phenotypes leading to elevated corticosterone, which is only observed on a 129, but not C57Bl/6J background (34).

### Mechanism of resetting of HPA axis feedback in C57Bl6 mice

The phenotype of HSD1^−/−^ mice originally described on the MF1/129 background, interpreted as reduced feedback sensitivity, is not surprising given the expression of HSD1 in feedback-related areas of the brain (19). The loss of local production of corticosterone would predict that higher plasma levels of hormone are required to reach equivalent tissue levels in feedback sites that normally express the enzyme. It is therefore of interest to speculate how C57Bl/6J HSD1^−/−^ mice maintain apparently normal HPA regulation. The elevation of GR expression in the hippocampus and PVN may well compensate for the lack of local cellular corticosterone regeneration. Indeed, the dexamethasone suppression test provides direct evidence for increased GR function because C57Bl/6J HSD1^−/−^ mice suppress evening corticosterone levels at a lower dose of dexamethasone than controls. This squares with the phenotype of transgenic mice with increased central nervous system (CNS) GR expression, which show suppressed basal HPA activity (39). Moreover, brain specific reduction of GR increases basal HPA activity (40), and global reduction of GR leads to prolonged HPA activity after restraint (41). Thus, the relative changes in central GR (increased in C57Bl/6J HSD1^−/−^ mice, and decreased in 129 or MF1/129 HSD1^−/−^ mice) correspond well with their phenotypes of relative high and low feedback, respectively. These differences suggest that the principle difference between strains may lie in the regulation of central GR expression. Indeed, similar HPA axis differences were observed when deletion of the neurokinin 1 receptor was investigated on a mixed 129/C57Bl/6 background compared to pure C57Bl/6, and, again, a strain dependent GR up-regulation was considered to underpin these changes (42). The mechanism by which elevated GR may occur in one strain, but not another, in response to ablation of HSD1 is unclear. Intriguingly, the GR gene uses a series of alternate promoters, several of which are CNS-enriched, and regulated by distinct transcription factors (43), so possibly this may underlie strain differences in the response to loss of 11β-HSD1. To elucidate the mechanisms underlying strain specific GR changes in response to a gene deletion, mapping of genetic modifiers using informative crosses of the relevant strains would need to be carried out. It is worth noting that GR was unchanged in the pituitary of C57Bl/6J HSD1^−/−^ mice relative to controls. A body of evidence indicates that the pituitary gland is an important site of action for glucocorticoid negative feedback (27, 44, 45). A lack of GR up-regulation in the pituitary in the C57Bl/6J HSD1^−/−^ mice strengthens our view that, in these experiments, feedback sensitivity is altered by GR changes in the brain itself. Hence, the correlation of GR mRNA up-regulation and increased glucocorticoid sensitivity is indicative of increased GR protein being expressed in the PVN and hippocampus, which comprise key sites of negative-feedback regulation of the HPA axis.

The results obtained in the present stusy, together with those from our previous reports (14, 21), suggest that 11β-HSD1 has a significant role in regulating the HPA axis. However, the manner of adaptation to the loss of 11β-HSD1 in genetically modified mice appears to be dependent upon other, probably genetic modifiers. The two phenotypes described on the C57Bl/6J and 129/MF1 backgrounds (i.e. either reduced or increased glucocorticoid receptor functioning) are potentially neuroendocrine markers of psychopathology. For example, melancholic depression is most commonly associated with elevated basal plasma glucocorticoids and impaired glucocorticoid receptor functioning (46, 47). By contrast, disorders such as post-traumatic stress disorder and atypical depression are associated with low or normal plasma steroids and increased glucocorticoid receptor functioning (46, 48). 11β-HSD1, therefore, may be an important factor in the overall regulation of the HPA axis in this clinical context, and may itself be relevant to disease susceptibility, severity or outcome. Furthermore, genetic modifiers of HPA adaptation to the loss of 11β-HSD1 may be important regulators of HPA axis function. In this regard, genetic dissection of 129 and C57Bl/6J mice strains could identify important genes involved in HPA regulation in health and disease. Inhibitors of 11ß-HSD1 are in development for metabolic and age-related cognitive disorders (43, 49). If the murine strain differences extrapolate to humans, then analysis of relevant human genotypes may allow the determination of sub-populations that may benefit from treatment with such agents without the unwanted side-effects of HPA axis dysregulation.
